# Comparison of Exendin-4 and Its Single Amino Acid Substitutions as Parent Peptides for GLP-1 Receptor Imaging Probes

**DOI:** 10.3390/molecules30051011

**Published:** 2025-02-21

**Authors:** Naoya Kondo, Maiko Yonezawa, Fuko Hirano, Takashi Temma

**Affiliations:** 1 Department of Biofunctional Analysis, Graduate School of Pharmaceutical Sciences, Osaka Medical and Pharmaceutical University, 4-20-1 Nasahara, Takatsuki 569-1094, Osaka, Japan; kondon@hirakata.kmu.ac.jp (N.K.); ompu72121051@s.ompu.ac.jp (F.H.); 2 Division of Fundamental Technology Development, Near InfraRed Photo-ImmunoTherapy Institute, Kansai Medical University, 2-5-1 Shin-machi, Hirakata 573-1010, Osaka, Japan

**Keywords:** GLP-1 receptor, exendin-4, nuclear medicine, peptide, insulinoma, blood glucose, diabetes

## Abstract

Glucagon-like peptide-1 receptor (GLP-1R) is an emerging critical target for the diagnosis and treatment of various diseases. Radiolabeled exendin-4 (Ex-4), a GLP-1R agonist, has been widely used as an imaging probe. However, its potential to induce hypoglycemia, especially in patients with insulinoma, limits its applicability. This study evaluated whether Ex-D3, a Glu3Asp substitution of Ex-4 with a higher internalization rate, could enhance the imaging efficacy of Ex-4 while reducing its hypoglycemic effects. We synthesized derivatives with an additional C-terminal Cys (Ex-D3-C40) for site-specific ^125^I labeling. Surface plasmon resonance analysis revealed that C-terminus modification did not significantly alter the binding affinity of Ex-D3-C40 to GLP-1R. In vivo studies in mice demonstrated that Ex-D3-C40 induced weaker hypoglycemic effects than Ex-4-C40. Biodistribution studies showed that ^125^I-labeled Ex-D3 ([^125^I]I-Ex-D3) achieved significantly higher pancreatic accumulation and higher pancreas-to-blood and pancreas-to-muscle ratios than [^125^I]I-Ex-4. Ex vivo autoradiography confirmed the binding specificity of [^125^I]I-Ex-D3 to GLP-1R-expressing pancreatic β-cells. These findings indicate that Ex-D3 is a promising parent peptide for the development of superior GLP-1R imaging probes with reduced hypoglycemic risk, highlighting the importance of considering pharmacological effects in designing molecular imaging probes.

## 1. Introduction

Glucagon-like peptide-1 receptor (GLP-1R) is crucial in glucose homeostasis and is expressed in various tissues throughout the body [1,2]. It is essential to the functioning of pancreatic β-cells, in which it is activated under hyperglycemic conditions to stimulate insulin secretion and thus helps regulate blood glucose levels [3,4]. This crucial function of GLP-1R has led to the successful development and commercialization of GLP-1 receptor agonists (GLP-1RAs) as effective treatments for diabetes [5,6]. Numerous probes for GLP-1R imaging have been developed by conjugating radioisotopes to GLP-1RAs based on their high binding affinity for GLP-1R, especially exendin-4 (Ex-4) [7,8,9].

This approach has been especially valuable in the identification of certain pathological conditions, with insulinoma emerging as a prominent theragnostic target [10,11,12]. The overexpression of GLP-1R in insulinoma cells makes it an ideal candidate target for molecular imaging [13,14]. ^68^Ga-NOTA-Ex-4 positron emission tomography–computed tomography (PET/CT) has demonstrated a sensitivity of 97.7% in detecting insulinoma, surpassing conventional imaging modalities [15]. Furthermore, in diabetes, chronic hyperglycemia has been associated with reduced GLP-1R expression in pancreatic β-cells, resulting in reduced insulin secretion [16]. This finding indicates that detecting changes in pancreatic GLP-1R expression could facilitate the early functional diagnosis of diabetes [2,17,18].

Despite these advancements, the use of peptide-based imaging probes poses challenges, particularly in separating radiolabeled peptides from their precursor peptides. Typically, the precursor peptide is included in the dosing solution with a controlled specific activity [19]. For GLP-1R probes, however, the precursor peptide itself acts as a potent GLP-1RA, posing hypoglycemic risks. For example, ^68^Ga-DOTA-Ex-4 can pose risks of hypoglycemia in patients with insulinoma, sometimes requiring glucose infusion during diagnosis [20,21,22]. Consequently, GLP-1RAs that exhibit a lower hypoglycemic effect during nuclear medicine procedures while maintaining high imaging efficacy need to be developed.

This study focuses on the relationship between the therapeutic efficacy of GLP-1RAs and their internalization rate. Upon binding to GLP-1R, GLP-1RAs undergo β-arrestin-mediated internalization [23]. Interestingly, the extent of internalization is inversely correlated with the extent of signaling: The longer a GLP-1RA binds to GLP-1R, the higher the internalization rate, which attenuates hypoglycemic effects [24,25]. From a therapeutic perspective, compounds that are less prone to internalization demonstrate superior glycemic control. Conversely, in nuclear medicine, imaging probes must have high target-specific accumulation and favorable background ratios with minimal pharmacological effects [26]. While various strategies have been employed to develop probes with high target accumulation, such as improving their blood retention and enhancing their affinity, cellular internalization remains a particularly crucial factor [27,28,29]. A probe internalized by the target cells requires more time for clearance than when it is merely bound to the surface of the receptors, resulting in the prolonged and specific retention of radioactivity in the target cells [30]. Rapid internalization has been observed in many clinically used peptide probes, such as DOTATATE [31,32]. Therefore, we hypothesized that GLP-1RAs with high internalization rates could serve as potent fundamental precursors of GLP-1R imaging probes with a lower probability of inducing hypoglycemia in nuclear medicine diagnostics. We investigated single amino acid substitutions of Ex-4, specifically the Glu3Asp substitution (Ex-D3), which is highly internalized into cells upon binding with GLP-1R [25] compared with the parent Ex-4, or His1Phe substitution (Ex-F1) which has a lower internalization capacity [25].

**Figure 1:**
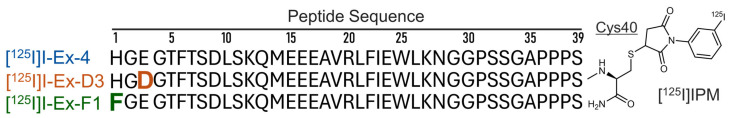
Schematic representation of radioiodinated exendin-4 derivatives labeled at the additional C-terminus Cys with [^125^I]IPM ([^125^I]Ex-4). [^125^I]Ex-D3 indicates the Glu3Asp substitution of [^125^I]Ex-4, while [^125^I]Ex-F1 indicates the His1Phe substitution at the N-terminus of [^125^I]Ex-4.

In this study, we synthesized Ex-4 derivatives with an additional Cys at the C-terminus (Ex-4-C40, Ex-D3-C40, and Ex-F1-C40) for radiolabeling. We evaluated their hypoglycemic activity and conducted Cys-specific ^125^I labeling studies using the Michael addition of maleimide and thiol ([^125^I]I-Ex-4, [^125^I]I-Ex-D3, and [^125^I]I-Ex-F1; Figure 1), employing the prosthetic agent *N*-(3-[^125I^]iodophenyl)maleimide ([^125^I]IPM) [33]. We evaluated the efficacy of single amino acid-substituted exendin-4 as a scaffold for GLP-1R imaging probes by examining the biodistribution of these labeled peptides in mice, especially focusing on Ex-D3 as a potentially superior parent peptide for GLP-1R imaging compared with Ex-4.

## 2. Results

### 2.1. Peptide Synthesis and Characterization

All peptides used in the experiments were synthesized with a purity of >99%. The electrospray ionization mass spectrometry (ESI-MS) data are shown in Table S1. The radiochemical yield for [^125^I]IPM was 72%, and it exhibited a radiochemical purity of >99%. High-performance liquid chromatography (HPLC) confirmed the complete separation of [^125^I]IPM from the precursor, with an estimated molar activity of 81 GBq/μmol, the same as the calculated molar activity of carrier-free ^125^I (Figure S1). The radiochemical yields for [^125^I]I-Ex-D3, [^125^I]I-Ex-4, and [^125^I]I-Ex-F1 were 43%, 54%, and 59%, respectively, all with a radiochemical purity >99%. Their HPLC retention times were 18.2, 17.7, and 18.5 min, respectively. HPLC analysis verified the successful separation of precursor peptides from labeled peptides, as no UV-detectable precursor contamination was observed in the purified labeled peptides (Figures S2 and S3).

The dissociation constant (K_D_) of each peptide to GLP-1R was determined by the surface plasmon resonance (Biacore), and is summarized in Table S2. The K_D_ values for Ex-D3, Ex-4, and Ex-F1 were 32.2, 33.6, and 24.1 nM, respectively, with no significant differences being observed among them (*p* > 0.05). For the C-terminal Cys-modified variants (Ex-D3-C40, Ex-4-C40, and Ex-F1-C40), the K_D_ values were 51.6, 36.0, and 31.8 nM, respectively, with no significant differences. The K_D_ values for the IPM-labeled I-Ex-D3, Ex-4, and I-Ex-F1 were 16.0, 28.1, and 36.6 nM, respectively, with no significant differences being observed. The addition of C-terminal Cys or IPM labeling did not cause any significant alterations in the K_D_ values compared with those of the corresponding parent peptides (*p* > 0.05 for all comparisons).

### 2.2. Glucose-Lowering Effects in Mice

**Figure 2:**
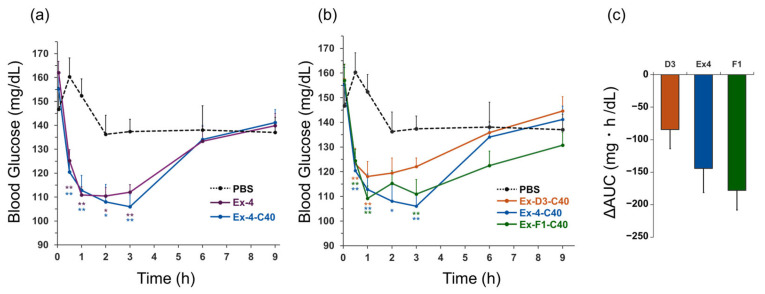
Comparison of the glucose-lowering effects of exendin-4 derivatives. (**a**) Blood glucose mg/dL in healthy mice (ddY, male, 38–42 g) after intravenous (i.v.) administration of (**a**) Ex-4, Ex-4-C40, or PBS; and (**b**) Ex-D3-C40, Ex-4-C40, Ex-F1-C40, or PBS. (**c**) ΔAUC (n = 12; 0–9 h) after i.v. administration of Ex-D3-C40 (D3), Ex-4-C40 (Ex4), or Ex-F1-C40 (F1) compared with PBS-treated control mice (n = 7). Dosage: 10 nmol/kg; formulation: 1 μM in PBS. Data are expressed as mean ± standard error of the mean and were analyzed by one-way analysis of variance with Dunnett’s multiple comparison test. * *p* < 0.05, ** *p* < 0.01, compared with PBS-administered control mice. ΔAUC, change in area under the curve.

The temporal changes in blood glucose levels following the intravenous administration of Ex-4, Ex-4-C40, and phosphate-buffered saline (PBS) are illustrated in Figure 2a, while Figure 2b presents the blood glucose levels for Ex-D3-C40, Ex-4-C40, Ex-F1-C40, and PBS. Comprehensive blood glucose levels are provided in Table S3. Statistical analysis revealed no significant differences between Ex-4 and Ex-4-C40 at any time point (Figure 2a). The blood glucose levels after Ex-D3-C40 administration tended to be higher than those for other peptide groups from 1 h after injection onward. The blood glucose reduction (change in area under the curve, ΔAUC) over 9 h for Ex-D3-C40, Ex-4-C40, and Ex-F1-C40 was −84, −144, and −178 mg·h/dL, respectively, with Ex-D3-C40 demonstrating the smallest reduction (Figure 2c).

### 2.3. Biodistribution

**Figure 3:**
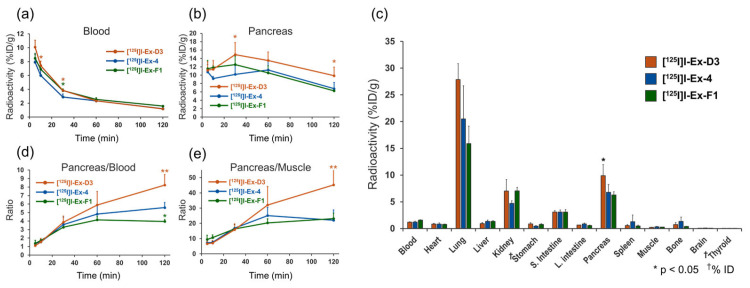
Biodistribution of [^125^I]I-Ex-D3, [^125^I]I-Ex-4, and [^125^I]I-Ex-F1 in healthy mice after intravenous administration. Radioactivity in the (**a**) blood and (**b**) pancreas. (**c**) Radioactivity distribution in internal organs at 120 min after administration. (**d**) Pancreas-to-blood and (**e**) pancreas-to-muscle radioactivity ratios. Data are expressed as mean ± standard deviation and were analyzed by one-way analysis of variance with Dunnett’s multiple comparison test. * *p* < 0.05, ** *p* < 0.01, compared with [^125^I]I-Ex-4.

The radioactivity accumulation data at 5, 10, 30, 60, and 120 min after the administration of [^125^I]I-Ex-D3, [^125^I]I-Ex-4, and [^125^I]I-Ex-F1 are presented in Table 1, Table 2, and Table S4, respectively. The three probes showed similar accumulation patterns in most organs. The blood radioactivity of [^125^I]I-Ex-4 was significantly lower than those of the other probes up to 30 min after administration, but became comparable thereafter (Figure 3a). The [^125^I]I-Ex-D3 accumulation in the pancreas, a GLP-1R-positive organ, was significantly higher than that of [^125^I]I-Ex-4 from 30 min after injection onward (Figure 3b). At 120 min after injection, the pancreatic radioactivity accumulation for [^125^I]I-Ex-D3, [^125^I]I-Ex-4, and [^125^I]I-Ex-F1 was 9.9%, 6.8%, and 6.3% injected dose per gram of tissue (%ID/g), respectively (Figure 3c). The pancreas-to-blood ratios were 8.2, 5.6, and 4.0, respectively, while the pancreas-to-muscle ratios were 45, 22, and 23, respectively (Figure 3d,e). High accumulation was also observed in the lungs, consistent with the previously reported high GLP-1R expression [34]. Among the three peptides, [^12^⁵I]I-Ex-D3 exhibited a tendency to have the highest accumulation in the lungs. Minimal thyroid accumulation was observed across all probes, indicating negligible in vivo deiodination.

**Figure 4:**
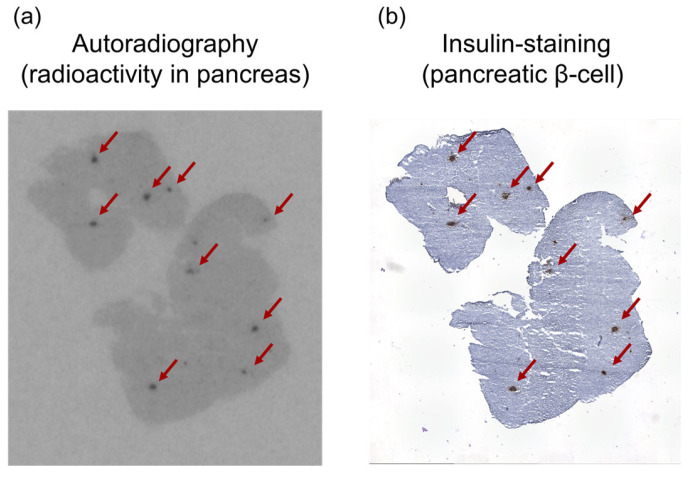
(**a**) Autoradiogram of [^125^I]I-Ex-D3 and (**b**) insulin immunohistochemical staining of representative pancreatic sections. Black spots indicate high radioactivity intensity in the autoradiogram, while brown-stained areas in the immunostaining image indicate insulin-positive regions. The red arrows in both images indicate the same position.

Ex vivo autoradiographic qualitative analysis of the pancreatic tissue following [^125^I]I-Ex-D3 administration revealed a distinct spot-like pattern of radioactivity localization (Figure 4a). This distribution pattern corresponded to insulin-positive regions in the immunohistochemically stained serial sections, confirming the specific targeting of pancreatic β-cells [35,36] (Figure 4b).

## 3. Discussion

This study evaluated the potential of Ex-D3, a Glu3Asp substitution of exendin-4 (Ex-4), as a parent peptide for GLP-1R imaging probes with a lower hypoglycemic effect. Ex-D3 can exhibit prolonged GLP-1R binding, higher internalization rates, and a lower insulin secretion capacity compared with Ex-4 [25]. Thus, we radiolabeled Ex-D3 while preserving its properties and assessed its efficacy for in vivo GLP-1R imaging.

We introduced a Cys residue at the C-terminus (C40) to facilitate specific radiolabeling while maintaining the crucial N-terminal conformation. Then, we conducted a surface plasmon resonance analysis of nine peptides (parent sequences, C40 adducts, and IPM-labeled forms) binding to immobilized GLP-1R to assess the impact of additional Cys and IPM labeling on the properties of the parent peptides. Remarkably, no significant differences in affinity were observed among the peptides, indicating that neither the addition of Cys nor IPM labeling substantially affected the peptides’ affinity to GLP-1R.

Blood glucose measurements showed that the addition of C-terminal Cys minimally impacted the hypoglycemic potential of Ex-4 (Figure 2a). Comparative analysis of the glucose-lowering ability of the three C40 adducts revealed similar initial effects. However, the blood glucose recovery tended to be faster with Ex-D3-C40, whereas Ex-F1-C40 prolonged the glucose-lowering effect (Figure 2b). The blood glucose ΔAUC indicated that the Ex-D3-C40 treatment caused less pronounced hypoglycemia compared with Ex-4-C40 or Ex-F1-C40 (Figure 2c), indicating that the C-terminal–modified Ex-D3 has a higher internalization capacity and lower hypoglycemic potential. Since the chemical forms remaining in the dosing solution after the reaction of the peptide precursors with carrier-free radionuclides are predominantly peptide precursors, we performed a comparative analysis of C40 adducts. In addition, we did not perform the experiments under glucose-loading conditions because we assumed a decrease in the blood glucose during nuclear medicine diagnosis. Future studies that incorporate glucose-loading conditions (e.g., intraperitoneal glucose tolerance test [24,37]) could further elucidate the glucose-lowering effects of peptides.

To test our hypothesis that a high internalization capacity results in better in vivo GLP-1R imaging, we comprehensively evaluated the radioactive accumulation in the pancreas after radioiodinated probe administration. GLP-1R is expressed in β-cells in the pancreas, and since the fraction of β-cells is less than 2% of all pancreatic cells [38], GLP-1R high-density localization occurs. Therefore, the specific accumulation of radioactivity due to GLP-1R can be evaluated in vivo by examining the amount and extent of the radioactive localization in the pancreas after the administration of the probe.

Notably, at 120 min after [^125^I]I-Ex-D3 administration, significantly higher pancreatic accumulation (9.9% ID/g) was achieved compared with the 6.8%I D/g achieved for [^125^I]I-Ex-4 (Figure 3b). The pancreas-to-blood and pancreas-to-muscle ratios of accumulated radioactivity, which are indicators of imaging contrast, were 8.2 and 45, respectively, surpassing the values of 5.6 and 22 obtained for [^125^I]I-Ex-4 (Table 1 and Table 2). Moreover, [^125^I]I-Ex-D3 exhibited a longer pancreatic accumulation over time. While the pancreas-to-blood and pancreas-to-muscle ratios remained stable for [^125^I]I-Ex-4, they continued to increase for [^125^I]I-Ex-D3 in the late phase (Figure 3d,e), indicating enhanced retention in GLP-1R-expressing cells. Ex vivo autoradiography revealed a highly localized, spot-like radioactivity distribution in the pancreas which corresponded to insulin-stained β-cells (Figure 4a,b). This finding confirms the ability of [^125^I]I-Ex-D3 to be used for GLP-1R-specific recognition in vivo. The high accumulation observed in GLP-1R-expressing lung tissues [34,39] further confirms the specificity of [^125^I]I-Ex-D3 for detecting GLP-1R in various tissues.

The similarity in kinetics among the three probes may be attributed to their single residue substitutions, particularly for Ex-D3 and Ex-4, where one carbon chain is shortened, indicating minimal changes in their GLP-1R-independent behavior in vivo (Figure 3c).

In this study, a simple precursor design employed the addition of Cys to the C-terminus of the parent peptide to allow labeling with maleimide. Ex-D3-C40 could be synthesized in the same manner as Ex-4, and the ^125^I labeling proceeded quickly and efficiently in a site-specific manner. The structural modification of the C-terminus by IPM did not significantly affect the binding affinities of the peptides to GLP-1R.

Although the clinical use of ^125^I radiotracers is limited, ^124^I-labeled or ^123^I-labeled derivatives of Ex-D3 could be adapted for PET or single-photon emission computed tomography [40]. Future studies should investigate the use of alternative radionuclides to evaluate the clinical potential of Ex-D3. Moreover, in other studies, Ex-4 was radiolabeled on Lys in its sequence [41,42], and further verification of the efficacy of Ex-D3 in these cases is also needed. Nevertheless, the Cys-maleimide labeling method is effective for labeling Ex-4 which does not contain Cys in its sequence and has been employed in many studies [8,43,44,45,46]. Therefore, Ex-D3-C40 is considered a potentially effective precursor peptide for radiolabeling.

This study was based on the high internalization capacity of Ex-D3 that was reported previously [25], and, as expected, [^125^I]I-Ex-D3 demonstrated long-term retention in GLP-1R-expressing pancreases. To determine the efficacy of [^125^I]I-Ex-D3, we compared the affinities Ex-4 derivatives using surface plasmon resonance analysis with immobilized GLP-1R, which revealed no significant differences among the peptides. A more detailed elucidation of their intracellular distribution and metabolism might provide a clearer evaluation of the differences between [^125^I]I-Ex-D3, [^125^I]I-Ex-4, and [^125^I]I-Ex-F1. However, the subcellular localization assessment of radioiodinated compounds remains technically challenging and is a subject for future research. The present study found that the high cellular retention of [^125^I]I-Ex-D3 in vivo, as indicated by its accumulation in the pancreas over time, and the autoradiographic confirmation of the specific binding of [^125^I]I-Ex-D3 to GLP-1R in vivo, collectively confirmed the potential of [^125^I]I-Ex-D3 for GLP-1R imaging. Ex-D3 alters the hypoglycemic property of Ex-4 without significantly changing its synthesis, radiolabeling, or in vivo behavior, demonstrating the potential of Ex-D3 as a highly effective replacement for current Ex-4-based probes, and offering significant improvements in diagnostic applications.

In conclusion, this study demonstrated that ^125^I-labeled Ex-D3 exhibited lower hypoglycemic potential than Ex-4 while achieving superior accumulation in GLP-1R-expressing tissues in vivo. The findings highlight the potential of Ex-D3 as a parent peptide for developing improved GLP-1R imaging probes with a reduced risk of hypoglycemia. Future studies should translate these findings to clinically relevant radionuclides and evaluate the performance of Ex-D3-based probes in various disease models, such as in insulinoma and diabetes. The results of this study also underscore the importance of considering pharmacological effects in the design of molecular imaging probes, providing valuable insights for improving probe development strategies.

## 4. Materials and Methods

### 4.1. Peptide Synthesis and Characterization

All reagents were obtained from commercial sources and used without further purification. The linear peptides were synthesized using the Fmoc solid-phase methodology using rink amide resin (A00213, Watanabe Chemical Industries, Ltd., Hiroshima, Japan). Peptides were cleaved from the resin using a mixture of trifluoroacetic acid (TFA)/phenol/water/triisopropylsilane (88/5/5/2, *v*/*v*/*v*/*v*). IPM was prepared as previously described [33]. Ex-D3-C40, Ex-4-C40, or Ex-F1-C40 were dissolved in PBS (pH 7.4) and added with IPM (1 eq.) to acetonitrile. The mixture was incubated for 30 min at room temperature to yield I-Ex-D3, I-Ex-4, and I-Ex-F1, respectively.

Peptide purification was performed using reversed-phase HPLC (RP-HPLC) using a COSMOSIL 5C18-AR-II column (10 ID × 250 mm, Nacalai Tesque, Kyoto, Japan) and detected by UV absorption (Shimadzu, Kyoto, Japan). The mobile phase consisted of a linear gradient of solvent A (0.1% TFA in water) and solvent B (0.1% TFA in acetonitrile), increasing from 90:10 to 40:60 (A:B, *v*/*v*) over a 25 min period at a flow rate of 5.0 mL/min. These conditions were employed in all experiments in this study.

Purified peptides were characterized by analytical HPLC under conditions identical to those used in semipreparative HPLC and by ESI-MS using an LCMS-8045 system (Shimadzu Corp., Kyoto, Japan). Acetonitrile was removed from the peptide solutions under reduced pressure, and residual water was eliminated via freeze-drying, yielding the purified peptides as white powder.

### 4.2. Surface Plasmon Resonance (SPR) Analysis

SPR analysis was performed using a Biacore T200 system (GE Healthcare, Tokyo, Japan). Recombinant human GLP-1R protein (0.5 μM, 13944-H02H; Sino Biological, Beijing, China) was immobilized on a CM5 sensor chip. Peptides were injected at four-fold serial dilutions (0, 0.1–100 nM) onto the chip. The resulting sensorgrams (n = 3 for each concentration) were fitted to a 1:1 interaction model using the Biacore analysis software to determine the dissociation constants (K_D_).

### 4.3. Glucose-Lowering Effects in Mice

Male ddY mice (Japan SLC, Shizuoka, Japan) were housed under a 12 h light/dark cycle with free access to food and water. All animal experiments were conducted in accordance with the institutional guidelines for animal experiments. The study protocol was approved by the Institutional Experimental Animal Committee (Permission Numbers: 21–76 and 22–76). The minimum required sample size for the study was determined using G Power software (version 3.1.9.7, Universität Düsseldorf, Nordrhein-Westfalen, Germany). The parameters for the calculation included a two-tailed test, an effect size of 2, a significance level (α) of 0.05, and a statistical power (1−β) of 0.8. Based on this analysis, the required sample size was calculated to be six animals per group. To ensure unbiased results, the experiment was conducted in a blinded manner. Specifically, drug administration and blood glucose measurements were performed by separate investigators to prevent potential bias.

The mice (n = 55, 9 weeks old, 38–42 g) were randomly assigned into groups (n = 12 for peptide treatments; n = 7 for control). The mice were administered with PBS(-) or peptides (10 nmol/kg) via intravenous injection (formulation: 1 μM in PBS). Blood glucose levels were measured before dosing and at 0.5, 1, 2, 3, 6, and 9 h after injection using a blood glucose meter (GA-3, Changsha Sinocare Inc., Changsha, China). The amount of blood glucose reduction induced by peptide administration was calculated using the following formula using the area under the curve (AUC) values:

ΔAUC = AUC_0–9h_ (each peptide) − AUC_0–9h_ (PBS, average, n = 7).

### 4.4. Radiolabeling

Na [^125^I]I (NEZ033L) was obtained from PerkinElmer Japan (Kanagawa, Japan). [^125^I]IPM was synthesized with slight modifications to previously reported methods [33]. Briefly, *N*-[*m*-Tri(n-butyl)stannylphenyl]maleimide (100 μg) was dissolved in methanol containing 5% acetic acid (100 μL) in an Eppendorf tube. Na [^125^I]I (20–30 MBq in NaOH aqueous solution) and *N*-Chlorosuccinimide (30 μg in 60 μL methanol) were added, and the reaction mixture was incubated at room temperature for 30 min. The product was purified via RP-HPLC, passed through a C18 Sep-Pak cartridge (Waters Corp., Milford, MA, USA), washed with water, and eluted with acetonitrile (15–20 MBq/100 μL).

For radiolabeling peptides, [^125^I]IPM in acetonitrile was reacted with Ex-D3-C40, Ex-4-C40, or Ex-F1-C40 (100 μg dissolved in PBS) for 30 min at room temperature. The reaction mixtures were purified using RP-HPLC under the same conditions applied to non-radioactive peptides. The purified radiolabeled peptides were further analyzed by RP-HPLC to confirm radiochemical purity, ensuring that their retention times matched those of their non-radioactive counterparts. The collected solutions of purified radiolabeled peptides were dried under reduced pressure. Peptides for in vivo experiments were reconstituted in 0.1% Tween 80 containing PBS (PBS-T_80_).

### 4.5. Biodistribution Study

The mice (n = 60, 5 weeks old, 27–30 g) were randomly assigned into groups and were fasted for 6 h prior to the intravenous administration of [^125^I]I-Ex-D3, [^125^I]I-Ex-4, or [^125^I]I-Ex-F1 (18.5 kBq/100 μL of PBS-T_80_). Mice were euthanized at 5, 10, 30, 60, or 120 min after treatment (n = 4 for each time point). The brain, heart, lungs, liver, pancreas, spleen, stomach, small intestine, large intestine, kidneys, muscle, skin, and bone were collected. The weight and radioactivity of each organ were measured using a NaI well-type scintillation counter (1470 Wizard; PerkinElmer Japan), and the %ID/g was calculated. The minimum required sample size for this study was determined using G Power software ver.3.1.9.7. The parameters for the calculation included a two-tailed test, an effect size of 2.5, a significance level (α) of 0.05, and a statistical power (1−β) of 0.8. Based on this analysis, the required sample size was calculated to be four animals per group. To ensure unbiased results, the experiment was conducted in a blinded manner. Specifically, drug administration and radioactivity measurements were performed by separate investigators to prevent potential bias.

### 4.6. Ex Vivo Autoradiography and Immunohistochemistry

The mice intravenously administered [^125^I]I-Ex-D3 (370 kBq/100 μL PBS-T_80_) were euthanized 60 min after the administration. Pancreatic tissues were excised and immediately frozen at −80 °C. The frozen tissues were sectioned into 10 μm slices using a microtome (Shiraimatsu Co., Ltd., Osaka, Japan). The sections were exposed to an autoradiography film (Amersham Hyperfilm MP, Cytiva, Tokyo, Japan) for five days. Developed films were scanned using a Corefido MC843 scanner (Oki Electric Industry, Tokyo, Japan). The adjument sections were fixed with acetone at −20 °C, washed with PBS containing 0.1% Tween 20 (PBS-T_20_), and blocked with Blocking One Histo (Nacalai Tesque) for 10 min. The primary antibody, anti-insulin antibody (2 μg/mL; ab181547, Abcam, Cambridge, UK), was diluted in PBS-T_20_ with 1.5% bovine serum albumin and incubated with the sections overnight at 4 °C. After rinsing with PBS-T_20_, the sections were incubated with EnVision+ System-HRP Labeled Polymer (Dako K4002, Agilent Technologies, Santa Clara, CA, USA) for 30 min at room temperature, followed by incubation with 3,3′-diaminobenzidine-hydrogen peroxide (Wako Pure Chemical Industries, Osaka, Japan) for 10 min. Sections were counterstained with hematoxylin. Bright-field images were obtained using a BZ-X810 microscope (Keyence Co., Osaka, Japan) and analyzed using a BZ-X800 Analyzer (Keyence Co.).

### 4.7. Statistical Analysis

Data are expressed as mean ± standard deviation or mean ± standard error of the mean. Statistical analyses were performed using one-way analysis of variance with Dunnett’s multiple comparison test on GraphPad Prism 8 (GraphPad Software, Boston, MA, USA). Differences were considered statistically significant at the 95% confidence level (*p* < 0.05) unless otherwise noted.

## Figures and Tables

**Table 1:** Biodistribution of radioactivity after the administration of [^125^I]I-Ex-D3 (% injected dose per gram tissue).

	Time After Administration				
	5 min	10 min	30 min	60 min	120 min
Blood	10.0 ± 1.0	7.4 ± 0.6	3.9 ± 0.1	2.3 ± 0.3	1.2 ± 0.1
Heart	4.0 ± 0.4	3.6 ± 0.3	1.9 ± 0.2	1.3 ± 0.2	0.8 ± 0.1
Lung	29.6 ± 9.8	36.2 ± 10.2	37.8 ± 10.5	32.6 ± 4.4	27.9 ± 3.0
Liver	3.5 ± 0.3	4.5 ± 0.6	2.8 ± 0.7	1.7 ± 0.2	0.9 ± 0.2
Kidneys	39.2 ± 13.5	43.7 ± 6.1	19.7 ± 1.8	17.4 ± 4.6	7.0 ± 2.2
Stomach ^¶^	1.3 ± 0.2	1.5 ± 0.2	1.4 ± 0.4	1.0 ± 0.2	0.9 ± 0.2
Small intestine	3.0 ± 0.4	2.9 ± 0.4	3.0 ± 0.4	3.6 ± 0.5	3.1 ± 0.3
Large intestine	1.2 ± 0.2	1.2 ± 0.1	1.0 ± 0.3	0.7 ± 0.1	0.6 ± 0.0
Pancreas	11.2 ± 2.0	11.4 ± 2.1	14.9 ± 2.8	13.5 ± 2.0	9.9 ± 2.0
Spleen	3.2 ± 0.4	2.4 ± 0.1	1.3 ± 0.3	0.8 ± 0.4	0.6 ± 0.1
Muscle	1.7 ± 0.1	1.6 ± 0.1	0.9 ± 0.1	0.4 ± 0.1	0.2 ± 0.0
Bone	2.3 ± 0.6	2.0 ± 0.5	0.9 ± 0.4	0.7 ± 0.3	0.7 ± 0.3
Brain	0.3 ± 0.0	0.3 ± 0.0	0.1 ± 0.0	0.1 ± 0.0	0.1 ± 0.0
Thyroid ^¶^	0.1 ± 0.0	0.1 ± 0.0	0.0 ± 0.0	0.0 ± 0.0	0.0 ± 0.0
Pancreas/Blood	1.1 ± 0.2	1.6 ± 0.4	3.9 ± 0.7	5.9 ± 1.6	8.2 ± 1.2
Pancreas/Muscle	6.6 ± 1.6	7.1 ± 1.7	15.9 ± 3.8	32.0 ± 12.2	45.2 ± 11.0

^¶^ Expressed as % injected dose. Data are expressed as mean ± standard deviation (n = 4).

**Table 2:** Biodistribution of radioactivity after the administration of [^125^I]I-Ex-4 (% injected dose per gram tissue).

	Time After Administration				
	5 min	10 min	30 min	60 min	120 min
Blood	7.9 ± 1.1	6.0 ± 0.8	2.9 ± 0.4	2.3 ± 0.1	1.2 ± 0.2
Heart	3.6 ± 0.4	2.7 ± 0.4	1.5 ± 0.2	1.3 ± 0.2	0.8 ± 0.2
Lung	30.2 ± 2.3	29.7 ± 9.0	32.3 ± 10.0	34 ± 8.1	23 ± 10.0
Liver	4.1 ± 0.5	4.4 ± 0.5	2.7 ± 0.5	2.4 ± 0.5	1.3 ± 0.3
Kidneys	33.3 ± 4.3	39.7 ± 4.5	16 ± 5.3	10.8 ± 2.2	4.7 ± 0.5
Stomach ^¶^	1.1 ± 0.2	1.1 ± 0.3	1.0 ± 0.3	0.9 ± 0.2	0.5 ± 0.1
Small intestine	2.1 ± 0.4	2.2 ± 0.2	2.9 ± 0.6	3.1 ± 0.5	3.1 ± 0.4
Large intestine	0.9 ± 0.1	0.9 ± 0.0	0.7 ± 0.2	0.7 ± 0.1	0.9 ± 0.2
Pancreas	10.8 ± 1.8	9.2 ± 0.5	10.2 ± 2.1	11.3 ± 2.3	6.8 ± 1.5
Spleen	2.2 ± 0.4	1.4 ± 0.3	0.9 ± 0.2	0.7 ± 0.1	1.3 ± 1.3
Muscle	1.5 ± 0.2	1.2 ± 0.1	0.6 ± 0.1	0.5 ± 0.1	0.3 ± 0.1
Bone	2.5 ± 0.2	1.9 ± 0.7	1.0 ± 0.3	1.2 ± 1.2	1.3 ± 0.8
Brain	0.4 ± 0.0	0.3 ± 0.0	0.1 ± 0.0	0.1 ± 0.0	0.1 ± 0.0
Thyroid ^¶^	0.1 ± 0.0	0.1 ± 0.0	0.0 ± 0.0	0.0 ± 0.0	0.0 ± 0.0
Pancreas/Blood	1.4 ± 0.1	1.5 ± 0.2	3.6 ± 0.9	4.8 ± 1.0	5.6 ± 0.6
Pancreas/Muscle	7.2 ± 0.7	7.6 ± 0.9	16.9 ± 2.2	25.0 ± 5.3	22.0 ± 6.7

^¶^ Expressed as % injected dose. Data are expressed as mean ± standard deviation (n = 4).

## Data Availability

The data supporting the results and findings of this study are available within the paper and the Supplementary Information files. Additional raw data are available from the corresponding author upon request.

## Supplementary Materials

The following supporting information can be downloaded at: www.mdpi.com/article/10.3390/molecules30051011/s1, Figure S1: Representative analytical HPLC chromatogram of [^12^⁵I]IPM; Figure S2: Representative semi-preparative HPLC chromatogram of the solution 30 min after reacting Ex-D3-C40 with [^125^I]IPM; Figure S3: Representative analytical HPLC chromatogram of the purified [^12^⁵I]I-Ex-D3; Table S1: ESI-MS data of synthetic peptides; Table S2: Dissociation constants of peptides to GLP-1R determined by Biacore; Table S3: Blood glucose data measured over time up to 9 h after peptide administration; Table S4: Biodistribution of radioactivity post-administration of [^125^I]I-Ex-F1.
