# Supplementary material for: Comparison of Exendin-4 and Its Single Amino Acid Substitutions as Parent Peptides for GLP-1 Receptor Imaging Probes
Source: Molecules. 2025 Feb 21;30(5):1011. doi: 10.3390/molecules30051011 (PMC11901735; doi:10.3390/molecules30051011)
Supplement: Supplementary file 1 [file molecules-30-01011-s001.zip › molecules-3478820-supplementary.pdf]

Supplementary Information for:

Comparison of exendin-4 and its single amino acid substitutions as parent peptides for GLP-1 receptor imaging probes

N. Kondo *et al.*

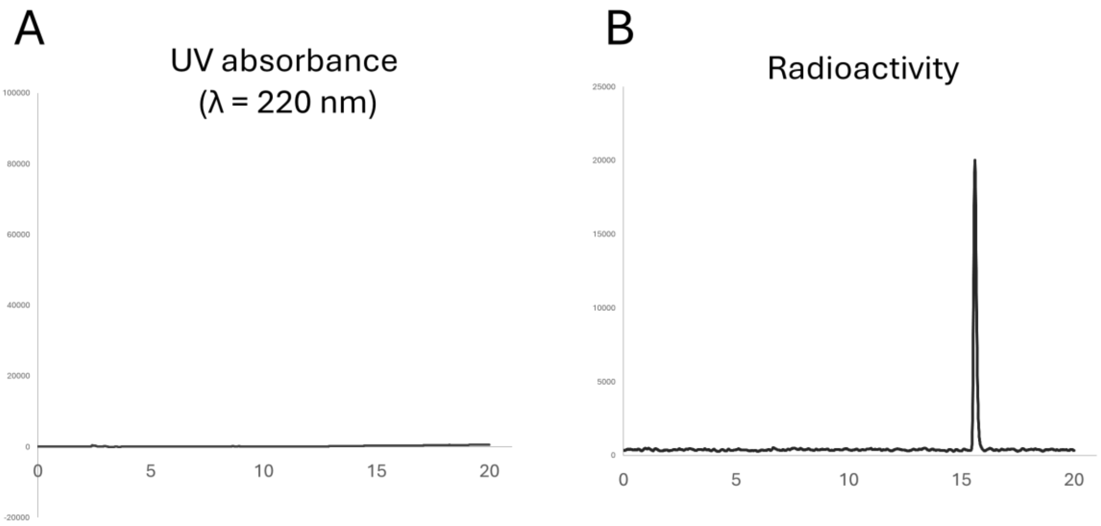

Figure S1

Representative analytical HPLC chromatogram of [^{125}I]IPM (A, UV absorbance; B, radioactivity). The mobile phase consisted of a linear gradient of solvent A (0.1% TFA in water) and solvent B (0.1% TFA in acetonitrile), increasing from 90:10 to 10:90 (A:B, v/v) over a 20-min period at a flow rate of 5.0 mL/min.

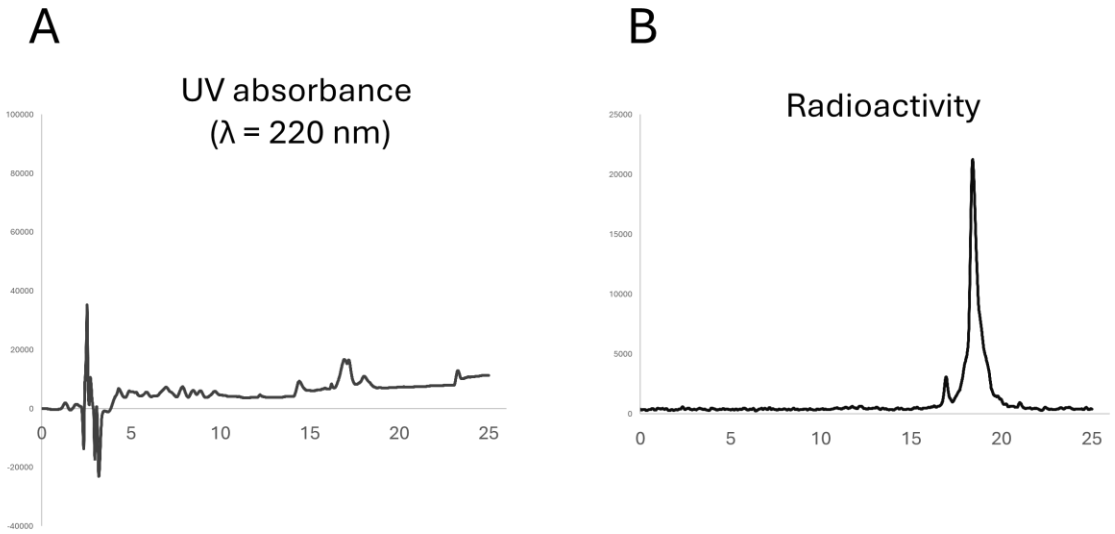

Figure S2

Representative semi-preparative HPLC chromatogram of the solution 30 min after reacting Ex-D3-C40 with [^{125}I]IPM (A, UV absorbance; B, radioactivity).

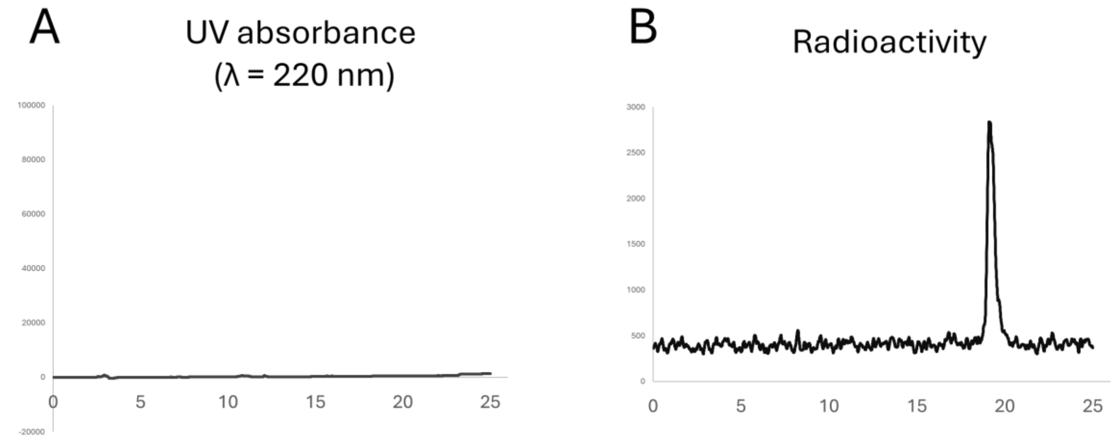

Figure S3

Representative analytical HPLC chromatogram of the purified[^{125}I]I-Ex-D3 (A, UV absorbance; B, radioactivity).

Table S1 ESI-MS data of synthetic peptides

	Chemical Formula	Calculated *m*/*z*	Detected *m*/*z*
Ex-4	$C_{184}H_{282}N_{50}O_{60}S$	4186	1396 $[M+3H^+]^{3+}$, 1047 $[M+4H^+]^{4+}$, 838 $[M+5H^+]^{5+}$
Ex-D3	$C_{183}H_{280}N_{50}O_{60}S$	4172	1391 $[M+3H^+]^{3+}$, 1044 $[M+4H^+]^{4+}$, 835 $[M+5H^+]^{5+}$
Ex-F1	$C_{187}H_{284}N_{48}O_{60}S$	4196	1400 $[M+3H^+]^{3+}$, 1050 $[M+4H^+]^{4+}$, 840 $[M+5H^+]^{5+}$
Ex-4-C40	$C_{187}H_{286}N_{50}O_{62}S_2$	4290	1431 $[M+3H^+]^{3+}$, 1073 $[M+4H^+]^{4+}$, 859 $[M+5H^+]^{5+}$
Ex-D3-C40	$C_{186}H_{284}N_{50}O_{62}S_2$	4276	1426 $[M+3H^+]^{3+}$, 1070 $[M+4H^+]^{4+}$, 856 $[M+5H^+]^{5+}$
Ex-F1-C40	$C_{190}H_{288}N_{48}O_{62}S_2$	4300	1434 $[M+3H^+]^{3+}$, 1076 $[M+4H^+]^{4+}$, 861 $[M+5H^+]^{5+}$
I-Ex-4	$C_{197}H_{292}IN_{51}O_{64}S_2$	4589	1530 $[M+3H^+]^{3+}$, 1148 $[M+4H^+]^{4+}$, 918 $[M+5H^+]^{5+}$
I-Ex-D3	$C_{196}H_{290}IN_{51}O_{64}S_2$	4575	1526 $[M+3H^+]^{3+}$, 1144 $[M+4H^+]^{4+}$, 916 $[M+5H^+]^{5+}$
I-Ex-F1	$C_{200}H_{294}IN_{49}O_{64}S_2$	4599	1534 $[M+3H^+]^{3+}$, 1151 $[M+4H^+]^{4+}$, 921 $[M+5H^+]^{5+}$

Table S2
Dissociation constants (K_D, nM) of peptides to GLP-1R determined by Biacore

	KD (nM)
Ex-4	33.6 ± 5.3
Ex-D3	32.2 ± 18.5
Ex-F1	24.1 ± 10.2
Ex-4-C40	36.0 ± 14.8
Ex-D3-C40	51.6 ± 11.9
Ex-F1-C40	31.8 ± 8.7
I-Ex-4	28.1 ± 18.5
I-Ex-D3	16.0 ± 4.9
I-Ex-F1	36.6 ± 21.4

Data are mean ± SD (n = 3).

Table S3

Blood glucose data (mg/dL) measured over time up to 9 hours after intravenous administration of peptides (10 nmol/kg)

	Ex-D3-C40	Ex-4-C40	Ex-F1-C40	Ex-4	PBS
Pre	157 ± 6	155 ± 7	157 ± 7	162 ± 5	147 ± 9
0.5 h	123 ± 6	120 ± 5	124 ± 5	125 ± 5	160 ± 8
1 h	118 ± 6	113 ± 6	109 ± 5	111 ± 3	152 ± 7
2 h	120 ± 6	108 ± 7	115 ± 5	110 ± 3	136 ± 8
3 h	122 ± 4	106 ± 5	111 ± 6	112 ± 3	137 ± 5
6 h	136 ± 5	134 ± 6	133 ± 6	133 ± 6	138 ± 10
9 h	145 ± 6	141 ± 5	140 ± 5	140 ± 5	137 ± 6

Data are mean ± SEM.

Table S4.
Biodistribution of radioactivity post-administration of [^{125}I]I-Ex-F1 (%ID/g)

	Time after administration (min)				
	5	10	30	60	120
Blood	8.5 ± 0.7	6.9 ± 0.5	3.8 ± 0.2	2.6 ± 0.3	1.6 ± 0.1
Heart	3.7 ± 0.1	2.8 ± 0.2	1.8 ± 0.1	1.0 ± 0.3	0.8 ± 0.1
Lung	26.2 ± 5.4	31.4 ± 1.5	26.0 ± 5.9	20.0 ± 2.9	15.9 ± 3.3
Liver	4.2 ± 0.2	4.0 ± 0.3	3.4 ± 0.5	2.1 ± 0.4	1.3 ± 0.1
Kidneys	27.0 ± 3.2	23.5 ± 3.7	19.0 ± 3.2	14.5 ± 4.4	7.1 ± 0.7
Stomach¶	1.2 ± 0.2	1.5 ± 0.1	1.0 ± 0.3	1.1 ± 0.3	0.8 ± 0.1
Small intestine	2.4 ± 0.0	2.5 ± 0.2	2.8 ± 0.4	2.9 ± 0.6	3.1 ± 0.4
Large intestine	1.1 ± 0.4	0.8 ± 0.1	0.8 ± 0.1	0.7 ± 0.1	0.6 ± 0.1
Pancreas	11.6 ± 2.0	11.8 ± 0.4	12.5 ± 2.0	10.6 ± 1.7	6.3 ± 0.6
Spleen	1.8 ± 0.3	1.3 ± 0.2	0.9 ± 0.1	0.5 ± 0.1	0.5 ± 0.2
Muscle	1.3 ± 0.2	1.1 ± 0.2	0.8 ± 0.0	0.5 ± 0.0	0.3 ± 0.0
Bone	2.3 ± 1.1	0.9 ± 0.1	0.8 ± 0.4	0.8 ± 0.6	0.4 ± 0.0
Brain	0.4 ± 0.1	0.3 ± 0.1	0.2 ± 0.0	0.1 ± 0.0	0.1 ± 0.0
Thyroid¶	0.1 ± 0.0	0.1 ± 0.0	0.0 ± 0.0	0.0 ± 0.0	0.0 ± 0.0
Pancreas/Blood	1.4 ± 0.4	1.7 ± 0.2	3.3 ± 0.4	4.1 ± 0.4	4.0 ± 0.2
Pancreas/Muscle	9.4 ± 2.8	10.7 ± 1.7	16.3 ± 2.7	20.3 ± 2.6	23.2 ± 3.5

¶Expressed as % injected dose. Data are mean ± SD (n = 4).
